# Supplementary figures and images for: Evaluating effectiveness of self-help groups in reduction of stigma in patients with neglected tropical diseases in Southern Nigeria: A cluster randomised study
Source: PLoS One. 2025 Oct 30;20(10):e0327741. doi: 10.1371/journal.pone.0327741 (PMC12574902; doi:10.1371/journal.pone.0327741)

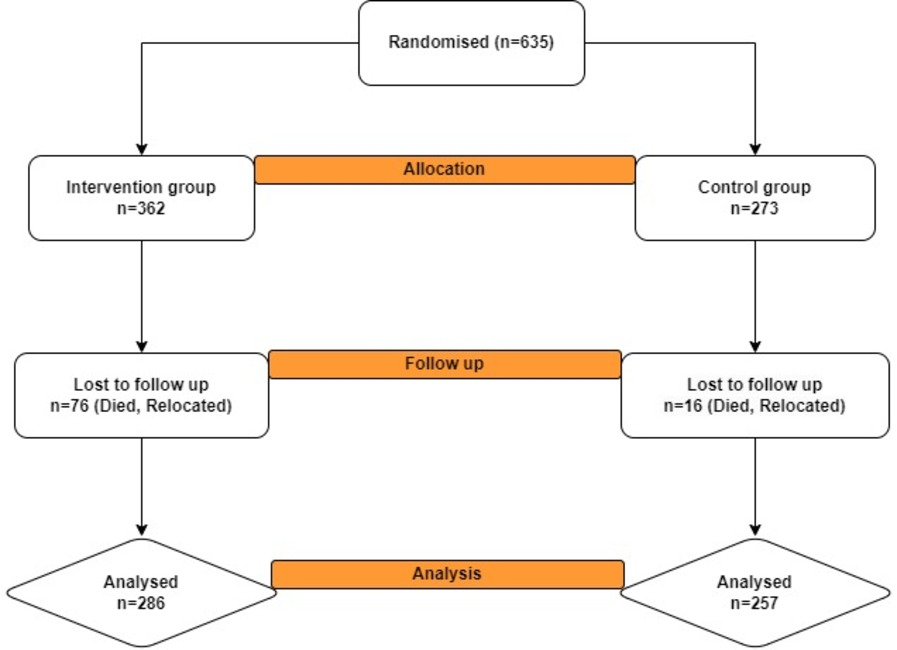

Supplement: S1 Fig — (TIF) [file pone.0327741.s003.tif]
