# Supplementary material for: Evaluating effectiveness of self-help groups in reduction of stigma in patients with neglected tropical diseases in Southern Nigeria: A cluster randomised study
Source: PLoS One. 2025 Oct 30;20(10):e0327741. doi: 10.1371/journal.pone.0327741 (PMC12574902; doi:10.1371/journal.pone.0327741)
Supplement: S1 Table — (DOCX) [file pone.0327741.s004.docx]

## S1 Table 1: Factors influencing the variability of scores at baseline

|  | **Pre-intervention** | | | |
| --- | --- | --- | --- | --- |
|  |  | **Coefficient (B)** | **p-value** | **95% Confidence Interval** |
| **Total Mean SARI Stigma score** | Constant | 31.24 | 0.000 | 28.37-34.12 |
|  | Intervention group | 10.75 | 0.000 | 8.05-13.46 |
|  | Buruli Ulcer | -8.47 | 0.000 | -11.57--5.37 |
|  | Employed | -10.83 | 0.000 | -13.51--8.16 |
| **Model: R Squared=0.232, p value<0.000, Residual: 244.02; Intercept variance: 202.01*** | | | | |
| **Internal Stigma Score** | Constant | 9.67 | 0.000 | 8.42-10.92 |
|  | Intervention group | 2.71 | 0.000 | 1.87-3.55 |
|  | Primary education | -1.10 | 0.023 | -2.04--0.16 |
|  | Higher education | -2.69 | 0.000 | -3.74--1.64 |
|  | Buruli Ulcer | -1.58 | 0.000 | -2.57--0.59 |
|  | Employed | -3.71 | 0.000 | -4.52--2.90 |
|  | Female | -1.14 | 0.000 | -1.91--0.37 |
| **Model: R Squared=0.268,p value<0.000, Residual: 22.24; Intercept variance: 0.0000*** | | | | |
| **Experienced Stigma Score** | Constant | 12.14 | 0.000 | 10.89-13.39 |
|  | Intervention group | 2.20 | 0.000 | 1.03-3.38 |
|  | Buruli Ulcer | -3.93 | 0.000 | -5.27--2.58 |
|  | Employed | -4.64 | 0.000 | -5.81--3.48 |
| **Model: R Squared=0.161, p value<0.000, Residual: 46.12; Intercept variance: 0.0000*** | | | | |
| **Disclosure Concern Score** | Constant | 5.24 | 0.000 | 4.51-5.96 |
|  | Intervention group | 3.45 | 0.000 | 2.77-4.13 |
|  | Buruli Ulcer | -2.34 | 0.000 | -3.12--1.55 |
|  | Employed | -1.05 | 0.000 | -1.73--0.38 |
| **Model: R Squared=0.193; p value<0.000, Residual: 15.58; Intercept variance: 5.79*** | | | | |
| **Anticipated Score** | Constant | 6.00 | 0.000 | 5.33-6.67 |
|  | Intervention group | 2.18 | 0.000 | 1.57-2.80 |
|  | Employed | -1.59 | 0.000 | -2.22--0.96 |
|  | Vocational training | 1.16 | 0.000 | 0.14-2.17 |
| **Model: R Squared=0.146; p value<0.000, Residual: 13.25; Intercept variance: 1.79** | | | | |

**^*Parameter was found to be redundant in the model, indicating that it did not contribute additional explanatory power^**
