# Supplementary material for: Evaluating effectiveness of self-help groups in reduction of stigma in patients with neglected tropical diseases in Southern Nigeria: A cluster randomised study
Source: PLoS One. 2025 Oct 30;20(10):e0327741. doi: 10.1371/journal.pone.0327741 (PMC12574902; doi:10.1371/journal.pone.0327741)
Supplement: S2 Table — (DOCX) [file pone.0327741.s005.docx]

## S2 Table: Factors influencing variability of scores post-intervention

|  | **Post intervention** | | | |
| --- | --- | --- | --- | --- |
|  |  | **Coefficient (B)** | **p-value** | **95% Confidence Interval** |
| **Total SARI Stigma score** | Constant | 42.35 | 0.000 | 40.74-43.97 |
|  | Intervention group | -37.72 | 0.000 | -39.43--36.01 |
|  | Employed | -2.16 | 0.013 | -3.86-0.46 |
|  | Vocational training | 2.71 | 0.005 | 0.80-4.62 |
| **Model: R Squared=0.78, p value=0.000, Residual: 97.48; Intercept variance: 0.0000*** | | | | |
| **Internal Stigma Score** | Constant | 8.06 | 0.000 | 7.57-8.54 |
|  | Intervention group | -7.58 | 0.000 | -8.1--7.07 |
|  | Employed | -0.70 | 0.006 | -1.20--0.20 |
|  | Buruli Ulcer | 1.16 | 0.001 | 0.60-1.73 |
|  | Vocational training | 0.55 | 0.049 | 0.00-1.10 |
| **Model: R Squared=0.62, p value=0.000, Residual: 8.07; Intercept variance: 2.03*** | | | | |
| **Experienced Stigma Score** | Constant | 16.63 | 0.000 | 16.07-17.19 |
|  | Intervention group | -15.44 | 0.000 | -16.21--14.67 |
| **Model: R Squared=0.74, p value=0.000, Residual: 20.95; Interception variance: 0.000*** | | | | |
| **Disclosure Concern Score** | Constant | 9.30 | 0.000 | 8.58-10.02 |
|  | Intervention group | -7.49 | 0.000 | -7.93--7.05 |
|  | Primary Education | -0.84 | 0.004 | -1.41--0.27 |
|  | Higher Education | -0.42 | 0.184 | -1.036-0.20 |
|  | Marital status_Married | -0.56 | 0.037 | -1.08--0.03 |
|  | Marital status_Others | 0.24 | 0.468 | -0.42-0.90 |
|  | Vocational training | 0.79 | 0.002 | 0.30-1.29 |
| **Model: R Squared=0.69, p value<0.000, Residual: 6.39; Intercept variance: 0.77** | | | | |
| **Anticipated Score** | Constant | 8.57 | 0.000 | 8.15-8.98 |
|  | Intervention group | -7.22 | 0.000 | -7.65--6.78 |
|  | Employment | -0.47 | 0.037 | -0.91--0.03 |
| **Model: R Squared=0.67, p value<0.000, Residual: 6.55; Intercept variance: 0.006*** | | | | |

**^*Parameter was found to be redundant in the model, indicating that it did not contribute additional explanatory power^**
