# Supplementary material for: Evaluating effectiveness of self-help groups in reduction of stigma in patients with neglected tropical diseases in Southern Nigeria: A cluster randomised study
Source: PLoS One. 2025 Oct 30;20(10):e0327741. doi: 10.1371/journal.pone.0327741 (PMC12574902; doi:10.1371/journal.pone.0327741)
Supplement: Attachment — Submitted filename: Rebuttal Letter.docx [file pone.0327741.s006.docx]

**Response to PLOS Neglected Tropical Diseases editor’s comment**

**Editor’s comment**: Although the research and the approach are commendable (and the larger study of which this is mentioned as a part of looks of major significance), there are differences in major parameters between intervention and control group.

**Response**: This study was a cluster randomized trial involving clusters across different regions of southern Nigeria. While observed differences between the study and control groups at baseline may not have been fully explained by the researchers, the authors employed appropriate statistical methods to address these differences during analysis (see the data analysis section). Specifically, they used a mixed-effects model for multivariate regression analysis, incorporating baseline covariates as predictors to adjust for potential imbalances. Additionally, the authors acknowledged several study limitations, which are also relevant to the interpretation of the findings.
