# Supplementary material for: Evaluating effectiveness of self-help groups in reduction of stigma in patients with neglected tropical diseases in Southern Nigeria: A cluster randomised study
Source: PLoS One. 2025 Oct 30;20(10):e0327741. doi: 10.1371/journal.pone.0327741 (PMC12574902; doi:10.1371/journal.pone.0327741)
Supplement: Attachment — Submitted filename: Response to Reviewers.docx [file pone.0327741.s007.docx]

**Response to reviewers’ comments**

Thank you for submitting your manuscript to PLOS ONE. After careful consideration, we feel that it has merit but does not fully meet PLOS ONE’s publication criteria as it currently stands. Therefore, we invite you to submit a revised version of the manuscript that addresses the points raised during the review process.

- confirm the exact number of states involved in the study: six or five. Single digits are better written in words to avoid typos.

Please include the following items when submitting your revised manuscript:

- A rebuttal letter that responds to each point raised by the academic editor and reviewer(s). You should upload this letter as a separate file labeled 'Response to Reviewers'.
- A marked-up copy of your manuscript that highlights changes made to the original version. You should upload this as a separate file labeled 'Revised Manuscript with Track Changes'.
- An unmarked version of your revised paper without tracked changes. You should upload this as a separate file labeled 'Manuscript'.

**************************************************************************************

Journal Requirements:

1. When submitting your revision, we need you to address these additional requirements.

Please ensure that your manuscript meets PLOS ONE's style requirements, including those for file naming. The PLOS ONE style templates can be found at

<https://journals.plos.org/plosone/s/file?id=wjVg/PLOSOne_formatting_sample_main_body.pdf> and

[https://journals.plos.org/plosone/s/file?id=ba62/PLOSOne_formatting_sample_title_authors_affiliations.pdf](https://track.editorialmanager.com/CL0/https:%2F%2Fjournals.plos.org%2Fplosone%2Fs%2Ffile%3Fid=ba62%2FPLOSOne_formatting_sample_title_authors_affiliations.pdf/1/010f01962186adbc-ac5a7591-8ee2-4e9f-bdd2-3b20ab56ba7f-000000/JTKX8jVP9eC1EcD3_HexNp2e2dos3-31hpMwZw2R0PI=206)

We confirm that the recommended file naming was used

2. Please include a complete copy of PLOS’ questionnaire on inclusivity in global research in your revised manuscript. Our policy for research in this area aims to improve transparency in the reporting of research performed outside of researchers’ own country or community. The policy applies to researchers who have travelled to a different country to conduct research, research with Indigenous populations or their lands, and research on cultural artefacts. The questionnaire can also be requested at the journal’s discretion for any other submissions, even if these conditions are not met. Please find more information on the policy and a link to download a blank copy of the questionnaire here: <https://journals.plos.org/plosone/s/best-practices-in-research-reporting>. Please upload a completed version of your questionnaire as Supporting Information when you resubmit your manuscript.

A completed version of the questionnaire is uploaded as a supporting document as requested. Thank you.

3. We note that Figure 1 in your submission contain [map/satellite] images which may be copyrighted. All PLOS content is published under the Creative Commons Attribution License (CC BY 4.0), which means that the manuscript, images, and Supporting Information files will be freely available online, and any third party is permitted to access, download, copy, distribute, and use these materials in any way, even commercially, with proper attribution. For these reasons, we cannot publish previously copyrighted maps or satellite images created using proprietary data, such as Google software (Google Maps, Street View, and Earth).

Thank you for your feedback regarding Figure 1. No proprietary data or satellite imagery from sources such as Google Maps, Earth, or Street View were used in the creation of this figure. The map is original and freely shareable under the terms required by PLOS.

I would like to clarify that the reference map was created entirely by us using QGIS, an open-source geographic information system. All map layers used were sourced from Global Administrative Areas (GADM) https://gadm.org/download_country.html, publicly available and open data, which are compatible with the Creative Commons Attribution License (CC BY 4.0).

4. Please review your reference list to ensure that it is complete and correct. If you have cited papers that have been retracted, please include the rationale for doing so in the manuscript text, or remove these references and replace them with relevant current references. Any changes to the reference list should be mentioned in the rebuttal letter that accompanies your revised manuscript. If you need to cite a retracted article, indicate the article’s retracted status in the References list and also include a citation and full reference for the retraction notice.

We confirm that the reference list have been reviewed and updated to accordingly.

Additional Editor Comments:

Line 117 - The number of states is five, not six. Single digits are better written in words to avoid typos.
Thank you. The correct number of states is six. This has been updated in the manuscript.

**Comments to the Author**

Review Comments to the Author

Please use the space provided to explain your answers to the questions above. You may also include additional comments for the author, including concerns about dual publication, research ethics, or publication ethics. (Please upload your review as an attachment if it exceeds 20,000 characters)

Reviewer #1: This is a commendable study and I congratulate the authors to their work. The results regarding the effectivity of the SHG intervention are truly impressive and I am aware of how much effort must have gone into organising and documenting the SHG in a study population of this size.

Thank you for your kind words. Its greatly appreciated.

The Introduction provides relevant background information about the stigmatisation of NTDs, especially leprosy.

The Methods section clearly explains the study design, and the information about statistical analysis is particularly detailed. However, it is unclear why children with BU are included but children with leprosy are excluded.

Thank you for this observation.

Owing to the epidemiology of leprosy in Nigeria, most persons affected are usually adults. This is quite unlike Buruli ulcer. In Nigeria, about 50% of persons affected by Buruli ulcer are children and young people. The researchers desired to include only participants up to 18 years and above. However, this may exclude a critical mass among those affected by Buruli ulcer. Hence, the need to include children affected by Buruli ulcer in the study.

However, during the implementation phase, a few (n=11) available child leprosy cases, mostly 10 years and above who were active participants in the Self-Help Groups (SHGs) were included. Their inclusion was driven by field realities, where SHGs naturally comprised individuals affected by leprosy regardless of age, and excluding them could have raised ethical concerns especially where their counterparts affected with BU were benefitting from the study. However, this had no impact on the overall analysis or validity of the study findings

Moreover, the qualitative aspects of the study (feedback from participants and SHG leaders) could be included both in the Methods and the Results section. They are brought up in the discussion and provide important insights into the participants' experience of stigma and of the self-help groups, so it looks like this is a mixed methods study rather than merely an analysis of the SARI scale. Please see also my more detailed comments in relation to specific parts of the manuscript (below).

Thanks for this valuable observation. While we agree that the feedback from participants and SHG leaders offers useful context and insights into participants' experiences, we do not consider this a mixed methods study, as the original design did not include a formal or structured qualitative data collection component. The narratives referenced in the discussion are reflective outputs from routine SHG meetings, where participants voluntarily shared experiences as part of the group process, rather than data collected through a defined qualitative methodology. We have clarified this in the methods and discussion sections of the manuscript to avoid any potential misinterpretation.

In the Data Collection section, I do not think that the other questionnaires need to be mentioned, as they are not presented in the manuscript. Instead, I would like to see more explanation about the SARI scale, including what type of questions were asked, what is the maximum score, and what the different scores mean (low/intermediate/high stigma?).

Thank you for this suggestion. We have now expanded the description of the SARI Stigma Scale in the Methods section to include more detail about the type of questions asked, the scoring and the interpretation. In addition, we have deleted information on the other questionnaires used that were not presented in the manuscript.

In the Results, the number of included children should be stated in the table on socio-demographics.

This has been included. Thank you.

I would also like to see information about visible disability, as this is presented as a major driver of stigma and can act as a confounder. It would also be important to see if there are differences in visible lesions/disability between the intervention and control group (which might explain the different levels of stigma at baseline). Presenting the relative reduction of stigma in the intervention group would highlight the effectiveness of the SHGs.

Thanks for this observation. We acknowledge this as a limitation in this study as we did not collect data on this key variable which could have provided more insight into stigma dynamics. However, this does not undermine the effectiveness of the intervention as the intervention group despite having higher stigma scores at baseline experienced a significant reduction in stigma post intervention, even if they potentially had higher levels of visible deformity.

The Discussion can be developed further by paying more attention to questions arising from the results: How do you explain the difference in stigma scores between the intervention and control group at baseline? And within the control group between pre- and post-intervention? Are these true differences or could they be an indication that the scale is not very reliable?

Thank you for this important observation. The difference in stigma scores between the intervention and control groups at baseline likely reflects real contextual and demographic differences between the groups rather than issues with the reliability of the SARI scale. As reported, participants in the intervention group had higher proportions of unemployment and lower levels of education and vocational training, which may contribute to increased vulnerability to stigma, particularly internalized stigma. These factors are known to influence stigma perception and experiences.

Furthermore, multivariate regression analysis revealed that being employed was significantly associated with lower stigma scores across all domains, while education was significantly associated with lower internalized stigma, supporting the interpretation that these baseline differences are meaningful and not measurement artifacts.

The within-group change in the control group between baseline and endline, highlights more the effectiveness of the intervention and suggests that without targeted support, stigma may worsen overtime.

The discussion section has been updated.

Line-by-line comments:
Line 72: consider cutting out “the infectious nature of NTDs” as not all NTDs are infectious and fear of contagion is already mentioned in the sentence. If you want to keep it because it relates to shame about having acquired the infection, then please change to “the infectious nature of most NTDs”

Thank you. We chose to keep it with the modification you suggested.

Lines 182-86: Why did you expect a prevalence of 50% and not 58% as in the Nigerian study? And why a reduction of 15% and not (over) 20% as in the Indian study?

Although the prevalence of 58% and >20% were used in Nigeria and India respectively, we opted for a more conservative estimate of 50% and 15% to remain grounded in the contextual realities of our study setting.

Lines 184/85: For context, please state that the study was from India

The manuscript has been updated with this.

Lines 202ff: Why did you include children with BU but not with leprosy?

Just as earlier described, there are peculiarities in the epidemiology of leprosy compared to BU in Nigeria. In Nigeria, most persons affected by leprosy are usually adults. The reverse is the case for Buruli ulcer.

In Nigeria, about 50% of persons affected by Buruli ulcer are children and young people. The researchers desired to include only participants up to 18 years and above. However, this may exclude a critical mass among those affected by Buruli ulcer. Hence, the need to include children affected by Buruli ulcer in the study.

Table 1: a) What exactly does “Diagnosis” mean? That they are affected by leprosy/BU or that they are currently under treatment? This is relevant to understand the drop in BU cases from pre- to post-intervention.

Thank you for pointing this out. Diagnosis in this context refers to their disease entity as persons affected by either leprosy or BU, regardless of their current treatment status. However, for clarity, we have revised the manuscript to replace diagnosis with “Disease entity”.

b) How many children were included in intervention and control group?

A total of 26 children were involved in the study: 18 in the intervention group and 8 in the control group.

c) A comparison of the prevalence of visible disability in the intervention and the control group would be useful. I understand that this is not socio-demographic data but important nevertheless, e.g. to understand the lower stigma rates in the control group pre-intervention, or why having a job is correlated with less stigma experience (with visible disability as confounder).

Please see the response above on the same question.

We acknowledge this as a limitation in this study as we did not collect data on this key variable which could have provided more insight into stigma dynamics. However, this does not undermine the effectiveness of the intervention as the intervention group despite having higher stigma scores at baseline experienced a significant reduction in stigma post intervention, even if they potentially had higher levels of visible deformity.

Lines 275ff: Can you summarise the (very impressive) impact of the intervention in a couple of sentences at the beginning of this section?

The manuscript has been updated with the summary

Line 330: …in the control group, with mean differences…

This has been corrected. Thank you.

Line 385: I think you can highlight here that your intervention had a very strong positive effect. It’s not only that the results were highly significant, but that stigma was reduced from 42% to 4.6% which is a truly impressive result showing that SHG are highly effective.

We are very grateful for your observation and kind words. The sentence has been revised accordingly.

Line 394: if you believe that the small reduction in internalized stigma in the control group is due to the ongoing leprosy awareness campaigns, how do you explain the overall increase in stigma in the control group?

We are not certain about what could have contributed to the slight change in internalized stigma. The manuscript has been revised accordingly to appropriately reflect our thoughts.

Line 398ff: This paragraph is very relevant and interesting as it discusses what makes the SHG so successful. However, the qualitative data this discussion is based on has not been presented in the results section; e.g. that “many felt emboldened to actively participate in social activities”. Including the feedback from the SHG leaders as a study result would make the paper stronger. Alternatively, a part of this paragraph could be moved to the Methods (explaining the objectives of the SHG in more detail) and the discussion here could be based more on the presented literature on HIV and mental health SHG.

Thank you once again for this. The methods and discussion sections of the manuscript have been updated to clarify this as explained earlier in this response document.

Line 424: Please clarify the statement that “being employed is associated with a reduction in total stigma”. Do you mean a reduction between pre- and post-intervention? Or do you mean that being employed is correlated with lower total stigma? Given that discrimination leads to not being employed, the bidirectional relationship between employment and stigma should at least be noted. In addition, visual disability is a potential confounder.

Our analysis found that being employed was associated with lower total stigma scores across most of the domains pre and post intervention, indicating a correlation rather than a pre-post reduction. However, it is noteworthy that the relationship between employment and stigma may be bi-directional, as stigma and discrimination can also negatively affect an individual’s employability, limiting their opportunities for social and economic participation. This has been highlighted in the revised manuscript.

Lines 451ff: If you want to discuss stigma reduction in children, you need to present the data in the results section. Children are not presented as a separate group and therefore there is no evidence of how your study offered insights into their challenges the effectiveness of the intervention in children.

Thanks for the observation. Since no subgroup analysis was conducted for children, the section referencing them has been removed from the discussion section to avoid misinterpretation.

Lines 453-456: as above, these observations (qualitative data) should be mentioned in the results section, if you use them in your discussion.

Has been clarified in the earlier responses and in the manuscript

Reviewer #2: General comments
The paper addresses an important topic which requires public health responses. The findings revealed could create an evidence base for policy and practice.

Specific comments
The paper could be improved if the authors address the following concerns
- The title needs some rephrasing as "...stigma in patients with neglected tropical diseases..."

Thank you very much. We agree with this submission and have updated the title.

- Abstract-methodology/principal findings section should clear elaborate the dimensions of stigma targeted by the intervention as well as the total number of samples in the intervention and control arms.

This section of the abstract has been updated with the relevant information as suggested. Thank you.

- In lines 76-79, the authors indicated sefl-stigma to be an important dimension of stigma with multiple negative consequences without justifying why their study should focus on this dimension of stigma for intervention in subsequent lines.

Thank you for the observation. While self-stigma is indeed an important aspect, particularly among persons affected by leprosy, the scope of this study extends beyond self-stigma. It encompasses other key dimensions of stigma, including experienced stigma, anticipated stigma, and disclosure concerns. Therefore, we did not consider it necessary to place additional emphasis on self-stigma alone in that section, as the intervention and analysis addressed multiple facets of stigma. However, we have revised that section for better clarity

- In methods section, there must be a separate section devoted for describing intervention outcomes. What are the intervention outcomes and how are they measured? Why is SARI tool selected among several other alternatives? What are the strengths, weaknesses and statistical attributes of the SARI tool? How is its validity and reliability determined in this study?

The primary outcome of the intervention was reduction in stigma among persons affected by leprosy or Buruli ulcer (BU). The four key domains of stigma were targeted namely- Internalized stigma, Anticipated stigma, Experienced stigma and Disclosure concerns. Changes in stigma were evaluated by comparing mean domain scores and total stigma scores between intervention and control groups at baseline and endline, using both within-group and between-group analyses.

The SARI Stigma Scale is a validated tool originally designed to measure perceived and experienced stigma among leprosy patients. However, research has shown that it is adaptable and applicable to other stigmatizing conditions, such as BU. This tool is best suited for our study as it measures multiple dimensions of stigma, offering a more comprehensive assessment than tools that focus only on self-stigma. It has also been previously used and validated in low-resource settings with populations similar to our study context, demonstrating good psychometric properties. For example, in a study conducted in Indonesia, the SARI Stigma Scale demonstrated good psychometric properties, with a Cronbach’s alpha of 0.88 for the total scale and intraclass correlation coefficient of 0.75, indicating strong internal consistency and reliability.

The scale consists of 21 items grouped into subdomains that capture key dimensions of stigma, including internalized stigma, perceived discrimination, disclosure concerns, and social exclusion. Each item is scored on a Likert scale from 0 for strongly disagree to 3 for strongly agree. The total score ranges from 0 to 63, with higher scores indicating greater levels of stigma.

The methods section of the manuscript has been updated accordingly. Thank you.

- It is also not clear for whom the SARI tool administered during evaluation. Were they members of the SHGs who participated in 24 months peer-support meetings? or Were they general category of leprosy and BU patients recruited from the sampled LGAs? If general patients, how were selected for pre-post evaluation? What was the relationship between SHGs and general category of patients during intervention implementation?

We had two groups, (Intervention and control groups). At baseline the SARI tool was administered to persons affected by leprosy or BU in the two groups. In the intervention group, SHGs were formed for persons affected by leprosy or BU and they participated in the peer-support meetings. The SARI tool was again administered to them post intervention. For the control group, the same tool was administered again to leprosy or BU persons in that group at the end of the study.

- The relevance of p-values in the table on line 272 is not clear. The table does not also make sense statistically.

The table presents the socio-demographic characteristics of participants in the intervention and control groups at baseline and at endline. The inclusion of p-values is intended to identify any statistically significant differences between the groups that could act as potential confounders in interpreting the effects of the intervention. This helps assess whether the groups were comparable at baseline and if any systematic differences emerged post-intervention that could influence the outcomes. However, potential confounders were controlled for in the multivariate regression analysis as described under the data analysis section.

6. PLOS authors have the option to publish the peer review history of their article ([what does this mean?](https://track.editorialmanager.com/CL0/https:%2F%2Fjournals.plos.org%2Fplosone%2Fs%2Feditorial-and-peer-review-process%23loc-peer-review-history/1/010f01962186adbc-ac5a7591-8ee2-4e9f-bdd2-3b20ab56ba7f-000000/06d7lXwLqqPa8-Yx_lRsYJ8O35-Xdws-uxhZoDVGXqM=206)). If published, this will include your full peer review and any attached files.


If you choose “no”, your identity will remain anonymous but your review may still be made public.


**Do you want your identity to be public for this peer review?** For information about this choice, including consent withdrawal, please see our [Privacy Policy](https://track.editorialmanager.com/CL0/https:%2F%2Fwww.plos.org%2Fprivacy-policy/1/010f01962186adbc-ac5a7591-8ee2-4e9f-bdd2-3b20ab56ba7f-000000/ngJjrDdsu1DaSz6A_dKUQ2WSmrFRLShY79LScIIKlYc=206).

Reviewer #1: No

Reviewer #2: No

[NOTE: If reviewer comments were submitted as an attachment file, they will be attached to this email and accessible via the submission site. Please log into your account, locate the manuscript record, and check for the action link "View Attachments". If this link does not appear, there are no attachment files.]

While revising your submission, please upload your figure files to the Preflight Analysis and Conversion Engine (PACE) digital diagnostic tool, [https://pacev2.apexcovantage.com/](https://track.editorialmanager.com/CL0/https:%2F%2Fpacev2.apexcovantage.com%2F/1/010f01962186adbc-ac5a7591-8ee2-4e9f-bdd2-3b20ab56ba7f-000000/jkJeCuv7f6zWEZmnx3qc9QHkNymGlG2AraI6VxlZ2OA=206). PACE helps ensure that figures meet PLOS requirements. To use PACE, you must first register as a user. Registration is free. Then, login and navigate to the UPLOAD tab, where you will find detailed instructions on how to use the tool. If you encounter any issues or have any questions when using PACE, please email PLOS at [figures@plos.org](mailto:figures@plos.org). Please note that Supporting Information files do not need this step.
