# Supplementary material for: Evaluating effectiveness of self-help groups in reduction of stigma in patients with neglected tropical diseases in Southern Nigeria: A cluster randomised study
Source: PLoS One. 2025 Oct 30;20(10):e0327741. doi: 10.1371/journal.pone.0327741 (PMC12574902; doi:10.1371/journal.pone.0327741)
Supplement: Attachment — Submitted filename: Response_to_Reviewers_auresp_2.docx [file pone.0327741.s008.docx]

**Reviewer’s comment and response from Authors**

**Reviewer comment**

Line 208: Do the authors have baseline data on the eleven children with leprosy? It seems the authors did not collect baseline data on few children with leprosy (n=11) but included them in the implementation phase for ethical concerns. If this is the case, the authors should explain this gap in the manuscript and acknowledge it as a limitation. Regarding the authors’ explanation in their response to reviewers’ comments, the reason, “Owing to the epidemiology of leprosy in Nigeria, most persons affected are usually adults,” is insufficient to exclude children with leprosy from participating in the study.

**Author response**

Thank you for your thoughtful observation. We would like to clarify that baseline and post-intervention data were indeed collected for all participants, including the eleven children affected by leprosy across the intervention and control groups.

As noted in the paper, this intervention was part of a larger randomised controlled study. While our original inclusion criteria for the study specified that children affected by leprosy would not be part of the study population, these children were identified during the community sensitization and mobilization activities. Given their status as persons affected by leprosy and in line with ethical principles of equity and beneficence, we felt it was important not to exclude them from benefiting from the intervention, particularly the self-help group component targeting stigma reduction-especially, since their counterparts affected by Buruli ulcer were part of the study.

Recognizing that this introduces a minor inconsistency between the inclusion criteria and implementation, we acknowledged this exception and its implication under the study limitation section of the manuscript.

We hope this clarification addresses your concern and we appreciate your commitment to ensuring the rigor and transparency of the study

**Reviewer comment**

Lines 187-191: The authors should explain the contextual realities in the study setting justifying the conservative estimate of 50% and 15% instead of the prevalence of 58% in Nigeria and at least 20% in India. In the absence of strong justification, the study should adopt the prevalence from the empirical evidence. The World Bank study, reference 21, is irrelevant to the sample size determination in this study and needs to be expunged.

**Author response**

Thank you once again for the valuable feedback. As previously noted, we were conservative while conceptualizing the study as we do not want to be ambitious by committing to the same prevalence and effect size especially where the contexts differ even within Nigeria. However, in line with your suggestion, we have updated our assumptions accordingly and confirm that our sample size remains adequate. In addition, we agree that the World Bank study added no value to the sample size determination and have removed it as advised.
