# Supplementary material for: Evaluating effectiveness of self-help groups in reduction of stigma in patients with neglected tropical diseases in Southern Nigeria: A cluster randomised study
Source: PLoS One. 2025 Oct 30;20(10):e0327741. doi: 10.1371/journal.pone.0327741 (PMC12574902; doi:10.1371/journal.pone.0327741)
Supplement: Attachment — Submitted filename: Response_to_Reviewers_auresp_3.docx [file pone.0327741.s009.docx]

**Editor’s comment and response from Authors**

**Additional Editor Comments:**

Generally, if data from a population outside the inclusion criteria for a study is collected, it should not be used unless there are specific ethical and methodological justifications. In the current study, it seems ethically inappropriate to have excluded children with leprosy from the study simply because leprosy was commoner among adults. The authors report that they identified 11 children with leprosy who met the inclusion criteria during community mobilisation, which is a sufficient reason to revise the study's inclusion criteria for people with leprosy. The authors further report that they collected baseline and post-intervention data from these 11 children with leprosy, and "their baseline and post-intervention data were collected and analysed in the same way as for other participants in the respective study groups." Excluding children with leprosy from the overall analysis after collecting pre- and post-intervention data from them is unethical and unacceptable. First, the study included children with Buruli ulcers. Secondly, the authors have an opportunity to revise the inclusion criteria. Thirdly, without reporting the findings on children with leprosy, we cannot ascertain whether excluding children with leprosy had any impact on the overall analysis or validity of the study. Therefore, the authors may revise the inclusion criteria and include the children with leprosy in the data analysis, results, and discussion.

**Authors’ response:**

Thank you very much for the valuable feedback. As recommended, we have revised the inclusion criteria to include child cases of leprosy. This is considered appropriate as the study already has ethical approval for child cases of BU and the contextual realities is such that children affected by leprosy are organically part of self-help groups. Excluding them is unethical as we acknowledged in our earlier response and you also highlighted it in your feedback. Moreso, the data collected from the 11 children affected by leprosy was included in the final analysis and results—pre and post-intervention—presented in the manuscript. However, there was no need to highlight them specially in the discussion section as block analysis and discussion was done for all age groups without particular focus on children.
